# Supplementary material for: Evaluation of risk adjustment performance of diagnosis-based and medication-based comorbidity indices in patients with chronic obstructive pulmonary disease
Source: PLoS One. 2022 Jul 8;17(7):e0270468. doi: 10.1371/journal.pone.0270468 (PMC9269939; doi:10.1371/journal.pone.0270468)
Supplement: S1 Table — (DOCX) [file pone.0270468.s001.docx]

**S1 Table. Results of binary logistic regression models with all covariates**

| **Variables** | | **2006 (n = 3,367)** | | | |  | **2009 (n = 3,191)** | | | |  | **2012 (n = 3,220)** | | | |
| --- | --- | --- | --- | --- | --- | --- | --- | --- | --- | --- | --- | --- | --- | --- | --- |
|  |  | **OR** | **95% CI** | | ***p*** |  | **OR** | **95% CI** | | ***p*** |  | **OR** | **95% CI** | | ***p*** |
| Gender (*reference: female*) | |  |  |  |  |  |  |  |  |  |  |  |  |  |  |
|  | The high-cost group | 1.53 | 1.29 | 1.81 | <.0001 |  | 1.54 | 1.29 | 1.83 | <.0001 |  | 1.13 | 0.95 | 1.34 | 0.1611 |
|  | In-hospital mortality | 2.37 | 0.50 | 11.16 | 0.2763 |  | 1.91 | 0.53 | 6.97 | 0.325 |  | 2.27 | 0.49 | 10.51 | 0.2957 |
| Age in years | |  |  |  |  |  |  |  |  |  |  |  |  |  |  |
|  | The high-cost group | 1.05 | 1.04 | 1.06 | <.0001 |  | 1.05 | 1.04 | 1.06 | <.0001 |  | 1.05 | 1.04 | 1.06 | <.0001 |
|  | In-hospital mortality | 1.07 | 1.01 | 1.14 | 0.0365 |  | 1.09 | 1.03 | 1.15 | 0.0052 |  | 1.10 | 1.03 | 1.17 | 0.0037 |
| If undergoing surgery (*reference: no*) | |  |  |  |  |  |  |  |  |  |  |  |  |  |  |
|  | The high-cost group | 3.97 | 3.10 | 5.08 | <.0001 |  | 4.20 | 3.21 | 5.50 | <.0001 |  | 4.75 | 3.64 | 6.19 | <.0001 |
|  | In-hospital mortality | 44.12 | 9.33 | 78.78 | <.0001 |  | 42.72 | 11.68 | 76.32 | <.0001 |  | 55.41 | 11.91 | 77.84 | <.0001 |
| Length of stay | |  |  |  |  |  |  |  |  |  |  |  |  |  |  |
|  | The high-cost group | 1.12 | 1.09 | 1.16 | <.0001 |  | 1.10 | 1.06 | 1.13 | <.0001 |  | 1.13 | 1.09 | 1.17 | <.0001 |
|  | In-hospital mortality | 1.04 | 1.02 | 1.06 | 0.0006 |  | 1.01 | 1.00 | 1.03 | 0.1261 |  | 1.05 | 1.02 | 1.08 | 0.0009 |
