# Supplementary material for: Evaluation of risk adjustment performance of diagnosis-based and medication-based comorbidity indices in patients with chronic obstructive pulmonary disease
Source: PLoS One. 2022 Jul 8;17(7):e0270468. doi: 10.1371/journal.pone.0270468 (PMC9269939; doi:10.1371/journal.pone.0270468)
Supplement: Attachment — Submitted filename: Response to Reviewers.pdf [file pone.0270468.s002.pdf]

Responses to the Comments of Dr. Jun Hyeok Lim, Academic Editor

1. Please ensure that your manuscript meets PLOS ONE's style requirements, including those for file naming.

Response: First and foremost, we would like to extend to Professor Lim our deep appreciation for your willingness to take over the editorship of our submitted manuscript which was submitted to *PLOS ONE* on June 3rd, 2021. In addition, we truly appreciate that you had assigned such qualified reviewers to our manuscript. Their thoughtful comments and constructive suggestions were a tremendous help to us during our revision process. Thank you so much.

As per your comment on our manuscript meeting *PLOS ONE*'s style requirements, including those for file naming, we have worked diligently to re-check our manuscript with respect to this advice for a couple of times, notwithstanding our manuscript had passed in-house technical check of the journal before. Thank you.

2. In your ethics statement in the Methods section and in the online submission form, please provide additional information about the data used in your retrospective study. Specifically, please ensure that you have discussed whether all data were fully anonymized before you accessed them and/or whether the IRB or ethics committee waived the requirement for informed consent. If patients provided informed written consent to have data from their medical records used in research, please include this information.

Response: Thank you for the reminder. In addition to the original relevant declaration under the Ethical approval section, we have included the following statements in the Methods of the revised manuscript:

"*Data in the NHIRD that could be used to identify patients or care providers, including medical institutions and physicians, are scrambled cryptographically and then released in electronic format to the public annually for research purposes by the National Health Research Institute of Taiwan. Since the present study utilized de-identified secondary data, it was exempt from full review by the Institutional Review Board of Taipei Medical University, Taiwan (TMU-JIRB No.*

N201605057)." (starting from the last paragraph of page 8).

3. Thank you for stating the following financial disclosure: "This study was supported by the Ministry of Science and Technology in Taiwan (grant number MOST 105-2410-H-038-010)."

Please state what role the funders took in the study. If the funders had no role, please state: "The funders had no role in study design, data collection and analysis, decision to publish, or preparation of the manuscript."
If this statement is not correct you must amend it as needed.
Please include this amended Role of Funder statement in your cover letter; we will change the online submission form on your behalf.

Response: We have added the following declaration to the Funding section of the revised manuscript and the cover letter as per your instructions. Thank you.

"*The funder had no role in study design, data collection and analysis, decision to publish, or preparation of the manuscript.*" (p. 18, 2nd main para).

4. In your Data Availability statement, you have not specified where the minimal data set underlying the results described in your manuscript can be found. PLOS defines a study's minimal data set as the underlying data used to reach the conclusions drawn in the manuscript and any additional data required to replicate the reported study findings in their entirety. All PLOS journals require that the minimal data set be made fully available. For more information about our data policy, please see http://journals.plos.org/plosone/s/data-availability.

Upon re-submitting your revised manuscript, please upload your study's minimal underlying data set as either Supporting Information files or to a stable, public repository and include the relevant URLs, DOIs, or accession numbers within your revised cover letter. For a list of acceptable repositories, please see http://journals.plos.org/plosone/s/data-availability#loc-recommended-repositories. Any potentially identifying patient information must be fully anonymized.

Important: If there are ethical or legal restrictions to sharing your data publicly, please explain these restrictions in detail. Please see our guidelines for more information on what we consider unacceptable restrictions to publicly sharing

data: http://journals.plos.org/plosone/s/data-availability#loc-unacceptable-data-access-restrictions. Note that it is not acceptable for the authors to be the sole named individuals responsible for ensuring data access.

Response: We appreciate your comments and suggestions. Actually, there are legal restrictions to sharing our data publicly. Here, we would like to re-state the related text under the Data availability statement subsection in our originally submitted manuscript, where we did declare that "(t)he Taiwan government prohibits release of the aforementioned datasets to the public domain":

"*The data underlying this study are from the National Health Insurance Research Database (NHIRD) which has been transferred to the Health and Welfare Data Science Center (HWDC). The Taiwan government prohibits release of the aforementioned datasets to the public domain. Interested researchers can obtain the data through formal application to the HWDC, Department of Statistics, Ministry of Health and Welfare, Taiwan (https://dep.mohw.gov.tw/DOS/np-2497-113.html).*" (p. 16, 2nd main para, the original manuscript; p. 19, 1st para, the revised manuscript).

5. Your ethics statement should only appear in the Methods section of your manuscript. If your ethics statement is written in any section besides the Methods, please move it to the Methods section and delete it from any other section. Please ensure that your ethics statement is included in your manuscript, as the ethics statement entered into the online submission form will not be published alongside your manuscript.

Response: As per your instructions, in addition to the original ethics statement under the Ethical approval subsection, we have included the following text in the Methods section of the revised manuscript:

"*Data in the NHIRD that could be used to identify patients or care providers, including medical institutions and physicians, are scrambled cryptographically and then released in electronic format to the public annually for research purposes by the National Health Research Institute of Taiwan. Since the present study utilized de-identified secondary data, it was exempt from full review by the Institutional Review Board of Taipei Medical University, Taiwan (TMU-JIRB No. N201605057).*" (starting from the last paragraph of page 8).

Responses to the Comments of Reviewer 1

1. The authors present a novel evaluation of model performance for predicting health care utilization in COPD based off administrative data. This is complementary to other previously-done evaluations, some of which are cited. The report is well written and clear. The methodology is logical, but leaves a few questions, which if answered, would strengthen the report.

Response: First and foremost, we want to extend our deep appreciation to Reviewer 1 for his/her encouraging remarks and excellent advice on our original submission. We have worked hard to be responsive to the reviewer's valuable comments. After addressing the significant issues raised by the reviewer, we feel that the quality of our manuscript is much improved, and hope the reviewer agrees.

Major comments:

1. Please explain your choice of years for inclusion in more detail. These data are now ≥9 years old. Was newer data not available? Was the choice related to the ICD-9 vs -10? If the latter, there are CCI and ECI algorithms available for ICD-10 as well.

Response: The reviewer had raised very focal points, and we are greatly appreciative of the opportunity for clarifications. While fully appreciating the fact of not using more recent datasets in this analysis as pointed out by the reviewer, we would also like to ask to consider some facts and limitations. Unfortunately, submission of this manuscript had encountered an unduly lengthy delay between our initial submission and the return of responses from journals. For example, before submitting our manuscript to *PLOS ONE*, we had submitted it to another journal. After nearly one year of delay (we had sent out inquiries to that journal for numerous times in between), we finally received a reviewing report where it stated succinctly that our paper was not a good fit for that journal's scope. Actually, we submitted our manuscript to *PLOS ONE* on June 3rd, 2021. We had sent out a couple of inquiries to the journal office of *PLOS ONE* with regard to the status of our submission, and the reply had always been that they had tried assiduously but had difficulty in finding an academic editor to handle our

submission (still, I would like to emphasize here that the staff at the journal office had always been acting promptly, professionally, and even empathetically in responding to our inquiries). Hence, we feel deeply grateful to Professor Lim for his willingness to take over the editorship of our submitted manuscript in the end, and truly appreciate that you would accept his invitation to be the reviewer of our manuscript and had provided us with thoughtful comments and constructive suggestions.

With that being said, we understand that this could not constitute a legitimate excuse for your question regarding our choice of data years for this analysis. We acknowledge that we do not utilize more updated datasets. The fact is that this study was supported by the Ministry of Science and Technology in Taiwan (grant number MOST 105-2410-H-038-010). At the time, the latest available data year for research purposes was 2012. There is a lag time of a couple of years as regards the Taiwan government releases the NHIRD. It is for research purposes only, and a relatively lengthy application procedure is required, including obtaining an IRB approval first. More important, we can access the dataset with a designated expiration date. Plus, if we intend to use more recent datasets, then we need to re-submit another application. At the time (i.e., year 2012) ICD-9-CM-codes were used (Taiwan's healthcare system switched to ICD-10-CM coding in 2016), albeit we recognize that the transition from ICD-9-CM-codes to ICD-10 is a momentously significant change in international disease coding in decades.

Although the data used in the study are relatively old (we totally agree with the reviewer's observation and opinion), we believe that the research topic is still relevant, and the trend and the pattern of data are mostly similar with respect to risk-adjusted comorbidities in patients with COPD. We hope the reviewer may agree.

2. Did you consider fitting a model with both RxRisk-V and ECI since they were the front-runners in model improvement?

Response: We appreciate this excellent suggestion from the reviewer. However, the objective of our study was to compare the performance of predicting medical expenditures and mortality in patients with COPD among various comorbidity indices. Hence, if we run a specific model comprising both RxRisk-V and ECI, it may pose challenge for us with regard to how to interpret the results in view of

comparisons with those results obtained from other models whereas only a single comorbidity index is included. Notwithstanding, your nice advice is well taken, and we may explore the issue in our future research. Thank you.

3. I don't see a clear comparison with statistical tests of significance of your model stability over the 3 time points. If these weren't done, I would recommend them; if they were done, please make it more clear in your reporting.

Response: We are grateful to the reviewer for giving us the opportunity for clarifications. We did not intend to compare statistical tests of significance of our model over the 3 time points. Rather, our intention was to carry out sensitivity analysis to validate the robustness of our results by repeating the analyses with additional target years (i.e., years 2006 and 2009). We had stated so in our manuscript: "*We further assessed the robustness of our models by repeating the analyses with three different target years; years 2006, 2009, and 2012.*" (p. 7, 1st main para, the original manuscript; p. 9, 1st main para, the revised manuscript). In addition, we had reported the related analytical results as well, although admittedly, the results were not quite perfect: "Specifically, the Elixhauser index added higher discriminatory capabilities when using the index hospitalization only as well as index and prior hospitalizations, compared with other comorbidity methods, in both year 2006 and year 2012." (starting from the last paragraph of page 9, the original manuscript; p. 12, 1st para, the revised manuscript). Thank you for your precious comment, again.

4. The discussion could be more detailed about the additive value of this investigation.

Response: We agree with the reviewer's observation. We have followed the reviewer's counsel and the revised and expanded text in the Discussion section is as follows:

"*Although some work has been done to compare diagnosis- and medication-based comorbidity indices (e.g., the Cortaredona study[39] in 2017), more research from different population and datasets is justified as to establish the respective merits of each comorbidity index and the generalizability of comparative performance. Viewed in this way, this study adds real-world evidence from population-based*

datasets and different data periods to the body of knowledge about the utility of various comorbidity indices. In particular, we have evaluated the relative performance of four diagnosis-based and two medication-based comorbidity indices with regard to two outcome measures of medical expenditures and in-hospital mortality of COPD patient altogether." (p. 12, 1st main para).

Reference cited in this reply and in the manuscript:

39. Cortaredona S, Pambrun E, Verdoux H, Verger P. Comparison of pharmacy-based and diagnosis-based comorbidity measures from medical administrative data. Pharmacoepidemiol Drug Saf. 2017; 26: 402-411. DOI: 10.1002/pds.4146 PMID: 27910177

Minor comments:

1. A prior evaluation of CCI versus ECI for utilization was omitted from the literature review. Consider citing Buhr RG et al, BMC Health Services. PMID 31615508.

Response: We are indebted that the reviewer would provide us with a nice reference which enriches our paper. The added text is as follows:

"Moreover, in their research focusing on patients with COPD, the same study population as the current investigation, Buhr and colleagues [56] concluded that the Elixhauser comorbidity index performed slightly better than the CCI in predicting the 30-day readmission risk." (p. 15, 1st main para).

Reference cited in this reply and in the manuscript:

56. Buhr RG, Jackson NJ, Kominski GF, Dubinett SM, Ong MK, Mangione CM. Comorbidity and thirty-day hospital readmission odds in chronic obstructive pulmonary disease: a comparison of the Charlson and Elixhauser comorbidity indices. BMC Health Serv Res. 2019; 19: 701. DOI: 10.1186/s12913-019-4549-4 PMID: 31615508

2. There's a typo on page 8, line 154, where "209" is presented instead of "2009"

Response: We are grateful for the reviewer's sharp eyes to have spotted our typo. We have made the required amendment on the last but three line of page10 of the revised manuscript. Thank you.

Responses to the Comments of Reviewer 2

1. The manuscript compares the improvement in prediction of outcomes in COPD at adding different comorbidity indices to a baseline prediction model. Results show that overall all comorbidity indices work well. themanuscript is clearly written in introduction and discussion. However, some aspects of Methods and Results require clarification and eventually additional work.

Response: First and foremost, we are very appreciative of Reviewer 2 for expending his/her precious time and energy to provide those detailed opinions and constructive feedback as well as positive comments. We found such insightful guidance tremendously helpful as we approached our revisions. We have earnestly attempted to be responsive to those salient observations and valuable suggestions of the reviewer. We hope that our responses and revisions meet with the reviewer's approval.

Major comments:

1. Objective. The title of the manuscript suggests that the different indices are assessed with respect to discrimination, although actually discrimination results (capacvity of the model to order individuals according to their risk of presenting the event) are not shown. This Reviewer suggests to conduct a real discrimination analysis or to change the objective (including title) according to the actual analysis.

Response: We appreciate the comment from the reviewer. The objective of our study was to assess and compare the added values of various comorbidity indices to the discriminatory/predicting ability regarding medical expenditures and mortality in patients with COPD, rather than focusing on individuals according to their risk of presenting the event (a person’s individual circumstances).

Upon reading the reviewer's opinion, however, we realize that our choice of term ("discriminative") in the manuscript title may cause misunderstanding, albeit the term has been used in other similar papers (e.g., the Kusumastuti et al. study). As per the reviewer's kind suggestions, we have replaced "discriminative" (and relevant terms) with "risk adjustment" in the manuscript title and throughout the manuscript. Accordingly, the revised manuscript title is: "*Evaluation of risk adjustment performance of diagnosis-based and medication-based comorbidity indices in patients with chronic obstructive pulmonary disease*" (the original title was: "*Evaluation of the discriminative power of diagnosis-based and medication-based comorbidity indices in patients with chronic obstructive pulmonary disease*"). Thank you.

Reference cited in this reply but not in the manuscript:

Kusumastuti S, Gerds TA, Lund R, Mortensen EL, Westendorp RGJ. Discrimination ability of comorbidity, frailty, and subjective health to predict mortality in community-dwelling older people: Population based prospective cohort study. Eur J Intern Med. 2017; 42: 29-38. DOI: 10.1016/j.ejim.2017.05.016 PMID: 28583408

2. Comorbidity indices. It would help the reader to understand a bit more on each of the indices. How were they selected? How many and which variables do they include? how does the scoring work? This information would help not only to understand the manuscript but mostly to take decisions based on your results.

Response: Thank you so much for pointing out the insufficient parts in our original manuscript. Based on the reviewer's comments, we have elaborated on descriptions of comorbidity indexes used in this paper as follows:

"*In the health services literature, widely used risk adjustment models based on coded comorbidities include the Charlson comorbidity index (CCI) [14], the Charlson/Deyo index [15], the Charlson/D'Hoore index [16], the Charlson/Romano index [17], and the Elixhauser index (EI) [18]. Those indices are based on a standard system for coding diagnoses, the International Classification of Diseases, Ninth Revision, Clinical Modification (ICD-9-CM) codes, from administrative health data of hospitalization or outpatient visit. The CCI was created by Charlson and colleagues [14] by using chart review to predict 1-year mortality in a cohort of 604 hospitalized patients in 1984. The*

index was revised in 1987 by including a list of 19 comorbid conditions, with each condition assigned a weight of 1, 2, 3, or 6, based on adjusted hazard ratios for each condition derived from Cox proportional hazards regression models. All of the individual weights were then added up to create a single comorbidity score for each patient. As for the Charlson/Deyo index, Deyo et al. [15] amended the CCI by identifying the ICD-9-CM diagnosis and procedure codes corresponding to each of the 19 comorbid conditions proposed by Charlson and colleagues. The codes for leukemia and lymphoma were combined in the 'any malignancy' category, and thus there was a list of 17 comorbid conditions for the Deyo CCI. As regards the Charlson/D'Hoore index [16], D'Hoore et al. adapted the CCI by using only the first three digits of ICD-9 coding without CM (since it is the coding fashion of many healthcare institutions outside the US). In addition, due to the likelihood that coding of the tailing digits in ICD-9 codes may lead to inconsistencies, therefore, D'Hoore et al. had declared that the Charlson/D'Hoore index was a more reliable comorbidity measure. The Charlson/Romano index [17], originally termed as the Dartmouth-Manitoba CCI, was firstly created by Roos et al. in 1989 and subsequently modified by Romano and colleagues in 1993. Compared with the Deyo CCI, the Romano CCI contains more ICD-9-CM codes. Concerning the Elixhauser index [18] was developed by Elixhauser and colleagues with a list of 30 comorbidities. In the literature there is strong evidence that the Elixhauser index outperforms the CCI, but the CCI continues to be widely used. One disadvantage of the EI is that unlike the CCI which produces a single comorbidity score on a continuous scale for each patient, the Elixhauser index entails 30 dichotomous variables but no weighting system to create a single score, making its use for analysis of comorbidity burdensome.

Another distinctive class of comorbidity instruments includes those indices using medication dispensing data to measure the burden of comorbid conditions; for example, the chronic disease score (CDS) [19], the modified chronic disease score (CDS-2) [20], the RxRisk index [21], and the RxRisk-V index [22]. The CDS, the first pharmacy-based measure of comorbidity, was created by von Korff and colleagues [19] in 1992. The methodology was based on medications rather than diagnostic codes to identify comorbid conditions of patients. A panel of experts was convened to evaluate patterns of utilization of selected medications as to create comorbidity categories, and weights were apportioned by consensus. The CDS consists of 17 comorbidity categories. Clark and colleagues [20] subsequently updated and modified the original CDS by expanding the disease categories to 28 as well as updating medications, and also assigned a weight to

each disease category based on results of regression models. With reference to the RxRisk index [21], it includes 57 disease categories and associated medication classes, and was originally developed as a risk assessment instrument by using outpatient pharmacy data to ascertain chronic diseases. As for the RxRisk-V index [22], it is a subsequent modification of the RxRisk-V index, consisting of 45 categories of comorbidity adapted to the United States Veterans Health Administration population." (pp. 5-7).

References cited in this reply and in the manuscript:

14. Charlson ME, Pompei P, Ales KL, MacKenzie CR. A new method of classifying prognostic comorbidity in longitudinal studies: development and validation. J Chronic Dis. 1987; 40: 373-383. DOI: 10.1016/0021-9681(87)90171-8 PMID: 3558716
15. Deyo RA, Cherkin DC, Ciol MA. Adapting a clinical comorbidity index for use with ICD-9-CM administrative databases. J Clin Epidemiol. 1992; 45: 613-619. DOI: 10.1016/0895-4356(92)90133-8 PMID: 1607900
16. D'Hoore W, Bouckaert A, Tilquin C. Practical considerations on the use of the Charlson comorbidity index with administrative data bases. J Clin Epidemiol. 1996; 49: 1429-1433. DOI: 10.1016/s0895-4356(96)00271-5 PMID: 8991959
17. Romano PS, Roos LL, Jollis JG. Adapting a clinical comorbidity index for use with ICD-9-CM administrative data: differing perspectives. J Clin Epidemiol. 1993; 46: 1075-1079; discussion 1081-1090. DOI: 10.1016/0895-4356(93)90103-8 PMID: 8410092
18. Elixhauser A, Steiner C, Harris DR, Coffey RM. Comorbidity measures for use with administrative data. Med Care. 1998; 36: 8-27. DOI: 10.1097/00005650-199801000-00004 PMID: 9431328
19. Von Korff M, Wagner EH, Saunders K. A chronic disease score from automated pharmacy data. J Clin Epidemiol. 1992; 45: 197-203. DOI: 10.1016/0895-4356(92)90016-g PMID: 1573438
20. Clark DO, Von Korff M, Saunders K, Baluch WM, Simon GE. A chronic disease score with empirically derived weights. Med Care. 1995; 33: 783-795. DOI: 10.1097/00005650-199508000-00004 PMID: 7637401
21. Fishman PA, Goodman MJ, Hornbrook MC, Meenan RT, Bachman DJ, O'Keeffe Rosetti MC. Risk adjustment using automated ambulatory pharmacy data: the RxRisk model. Med Care. 2003; 41: 84-99. DOI: 10.1097/00005650-200301000-00011 PMID: 12544546
22. Sloan KL, Sales AE, Liu CF, Fishman P, Nichol P, Suzuki NT, et al. Construction and characteristics of the RxRisk-V: a VA-adapted pharmacy-

based case-mix instrument. Med Care. 2003; 41: 761-774. DOI: 10.1097/01.MLR.0000064641.84967.B7 PMID: 12773842

3. Study subjects. Patients were selected based on having had an admission due to COPD. The information provided shows a specificity of 78%, which is not high. It is surprising that some patients undergo surgery during the index admission - which surgery would require a COPD admission (typically an exacerbation)? I suggest additional criteria are applied (availability of lung fucntion tests, some drugs treatment) to improve the validity of the COPD diagnosis.

Response: We truly appreciate such a valid point and insightful guidance relating to the study population of this analysis. In our manuscript, we stated that "(t)he study population comprised all patients who were hospitalized due to chronic obstructive pulmonary disease (COPD; ICD-9-CM codes: 491.x, 492.x, 496.x) for the first time in the target year of 2006, 2009, or 2012. The codes and algorithms had been validated and found to have a sensitivity of 85.0% and a specificity of 78.4% [33]." (*p. 7, 1st main para, the original manuscript*). While we entirely agree with the reviewer's opinion that a specificity of 78% may not be optimal enough, the same criteria had also been embraced by researchers in a relatively recent publication [40]. Pertaining to the reviewer's professional suggestions, information of lung function test results is not available in the NHIRD, whereas data concerning medications of patients with COPD were not among the requested data files when we applied for the secondary datasets, unfortunately. That said, those comments of specialty from the reviewer are greatly appreciated, again, as they are valuable guidance when we conduct relevant studies in the future. Thank you.

References cited in this reply and in the manuscript:

33. Gershon AS, Wang C, Guan J, Vasilevska-Ristovska J, Cicutto L, To T. Identifying individuals with physician diagnosed COPD in health administrative databases. COPD. 2009; 6: 388-394. DOI: 10.1080/15412550903140865 PMID: 19863368
40. Zhan ZW, Chen YA, Dong YH. Comparative performance of comorbidity measures in predicting health outcomes in patients with chronic obstructive pulmonary disease. Int J Chron Obstruct Pulmon Dis. 2020; 15: 335-344. DOI: 10.2147/COPD.S229646 PMID: 32103932

4. Sample size. The number of patients having a in-hospital death is very small. Was the study powered for it? If not, I suggest removing this part of the analysis.

Response: We agree with the reviewer's observation. While we are aware that low sample size will give rise to low statistical power, we did not determine the optimal sample size as to assure an adequate study power beforehand because this was a nationwide population-based study. As regards small numbers of patients having an in-hospital death, it is the fact in our study, admittedly. Be that as it may, COPD is well recognized as an important global public health challenge because it becomes a major and growing source of morbidity and mortality in countries at all levels of economic development [24,25]. Accordingly, in-hospital mortality is a prominent outcome measure in the relevant literature. For that reason, we would be very grateful if the reviewer could allow us to present our real-world data in this aspect. Thank you.

References cited in this reply and in the manuscript:

24. Global Initiative for Chronic Obstructive Lung Disease. Global Strategy for the Diagnosis, Management, and Prevention of Chronic Obstructive Pulmonary Disease. 2018. Available at: https://goldcopd.org/wp-content/uploads/2017/11/GOLD-2018-v6.0-FINAL-revised-20-Nov_WMS.pdf. Accessed May 20, 2021.
25. Mannino DM, Buist AS. Global burden of COPD: risk factors, prevalence, and future trends. Lancet. 2007; 370: 765-773. DOI: 10.1016/S0140-6736(07)61380-4 PMID: 17765526

5. Analysis, regression models. It is not clear how a logistic model is fitted for the outcome "expenditure", which is continuous in nature. Please clarify.

Response: We are truly grateful to the reviewer for bringing up a critical point that we neglected to describe explicitly in our original manuscript. Actually, both outcome measures (medical expenditures and in-hospital mortality) were dichotomous variables in this study, and that's why we conducted binary logistic regression analyses. Particularly pertaining to the outcome variable of medical expenditures which the reviewer was concerned, it was converted from a continuous variable to a dichotomous one using the threshold of the 75th

percentile (Q3), in consultation with the literature (Kadlec et al., 2013; van der Hulst et al., 2022). Indeed, that is the reason as regards we presented related descriptive statistics of Q3 in Table 1 of our original manuscript. We now elaborate on this vital issue under the Statistical analysis subsection as follows:

"Firstly, medical expenditure data revealed a positively skewed distribution, and thus they were converted to natural logarithm values. For all logistic regression models, medical expenditures were then dichotomized and the threshold was set at Q3 (the 75th percentile), with Q1-Q3 in the low-cost group whereas Q4 in the high-cost group, in accordance with previous research [34,35]." (starting with the last line of page 9).

References cited in this reply and in the manuscript:

34. Kadlec AO, Ellimoottil C, Guo R, Trinh QD, Sun M, Turk, TM. Contemporary volume-outcome relationships for percutaneous nephrolithotomy: results from the Nationwide Inpatient Sample. J Endourol. 2013; 27: 1107-1113. DOI: 10.1089/end.2013.0172 PMID: 23718230
35. van der Hulst M, Polinder S, Kok R, Prinzie P, de Groot MW, Burdorf A, Bertens LCM. Socio-economic determinants of healthcare costs in early life: a register-based study in the Netherlands. Int J Equity Health. 2022; 21: 5. DOI: 10.1186/s12939-021-01589-x PMID: 35022032

6. Analysis, discrimination. I suggest to add, for each index, the predicted outcome probability according to index scores, to assess (at least visually) discrimination.

Response: We appreciate this suggestion from the reviewer. We conducted related statistical analyses and constructed tables 2 and 3 with the current contents after consulting the relevant literature (e.g., Schneeweiss et al., 2001; Farley, Harley & Devine, 2006). To compare the performance of the Charlson, Elixhauser, RxRisk-V and other comorbidity indices, the *c*-statistics derived from multiple logistic regression models are typically used, as we had done so in our analysis as well. Upon reading the reviewer's suggestion, we have held extensive discussions, but still, we are not quite sure how we could make the statistics of the predicted outcome probability according to index scores fit in with the current tables 2 and 3. Moreover, we are concerned that the revised tables would become too multifaceted to be fittingly interpreted by the reader, then. While we support the referee's assertion that the information pertaining to the predicted outcome

probability according to index scores has its merit, we would appreciate it very much if our clarifications might be acceptable to the referee.

References cited in this reply and in the manuscript:

44. Schneeweiss S, Seeger JD, Maclure M, Wang PS, Avorn J, Glynn RJ. Performance of comorbidity scores to control for confounding in epidemiologic studies using claims data. Am J Epidemiol. 2001; 154; 854-864. DOI: 10.1093/aje/154.9.854 PMID: 11682368
58. Farley JF, Harley CR, Devine JW. A comparison of comorbidity measurements to predict healthcare expenditures. Am J Manag Care. 2006; 12: 110-119. PMID: 16464140

Minor comments:

1. I suggest to show the actual regression models with all their covariates as supplementary information.

Response: This is a good suggestion. As per the reviewer's advice, we have presented results of binary logistic regression models with all covariates as supplementary materials with the revised manuscript. Thank you very much.
