# Supplementary material for: Evaluation of risk adjustment performance of diagnosis-based and medication-based comorbidity indices in patients with chronic obstructive pulmonary disease
Source: PLoS One. 2022 Jul 8;17(7):e0270468. doi: 10.1371/journal.pone.0270468 (PMC9269939; doi:10.1371/journal.pone.0270468)
Supplement: Attachment — Submitted filename: Response to Reviewers.docx [file pone.0270468.s003.docx]

**Response to the Comment of Reviewer 1**

1. All comments have been addressed.

**Response:** We appreciate it very much that our responses and revisions meet with the reviewer's approval.

**Responses to the Comments of Reviewer 2**

1. THe question on statistical power is not irrelevant, I understand the sample size is what it is. However, the issue with low power is that results may be simply wrong (because one can not discard chance). I strongly suggest that the authors add a sentence/short paragraph with statistical power calculations (whatever the result - 10%, 40% or 99%) and let the reader interpret.

**Response:** We appreciate, and agree with, the viewpoint of the reviewer. As per the reviewer’s comments, we have performed statistical power calculations of this study as follows:

“*To characterize the power of this study, we employed the* [*PROC POWER*](https://documentation.sas.com/doc/en/pgmsascdc/9.4_3.4/statug/statug_power_syntax01.htm) *statement in the POWER procedure of the SAS/STAT software (SAS Institute, Cary, NC, USA). The computed study power* *was 0.824*.” (p. 12, last para, the revised manuscript).

Thank you very much for your valuable comment, again.

2. The description of the diverse indices is very useful but maybe too extended for the introduction and completely cuts the flow. I suggest to keep it short in introduction and detailed in methods.

**Response:** We are grateful to the reviewer for this very helpful and concrete suggestion. As per the reviewer’s advice, we have shortened the related text of descriptions of the diverse indices in the Introduction and placed detailed explanations of the indices in the Methods (we highlighted the revised text in yellow throughout the revised manuscript). Thank you.
